# COPD symptoms in the morning: impact, evaluation and management

**DOI:** 10.1186/1465-9921-14-112

**Published:** 2013-10-21

**Authors:** Nicolas Roche, Niels H Chavannes, Marc Miravitlles

**Affiliations:** 1 Respiratory and Intensive Care Medicine, Cochin Hospital Group, AP-HP University Paris Descartes, Paris, France; 2 Department of Public Health and Primary Care, Leiden University Medical Center, Leiden, Netherlands; 3 Hospital Universitari Vall d’Hebron, Servei de Pneumologia, Barcelona, Spain

**Keywords:** Morning symptoms, Onset of action, Once-daily, Long -acting muscarinic antagonists, Long-acting β_2_ agonists

## Abstract

Chronic obstructive pulmonary disease (COPD) symptoms in the morning, including dyspnea and sputum production, affect patients’ quality of life and limit their ability to carry out even simple morning activities. It is now emerging that these symptoms are associated with increased risk of exacerbations and work absenteeism, suggesting that they have a more profound impact on patients than previously thought. The development of validated patient-reported outcome (PRO) questionnaires to capture patients’ experience of COPD symptoms in the morning is, therefore, vital for establishing effective and comprehensive management strategies. Although it is well established that long-acting bronchodilators are effective in improving COPD symptoms, the limited available data on their impact on morning symptoms and activities have been obtained with non-validated PRO questionnaires. In this review, we discuss the impact of COPD symptoms in the morning and available tools used to evaluate them, and highlight specific gaps that need to be addressed to develop standardized instruments able to meet regulatory requirement. We also present available evidence on the effect of pharmacological therapies on morning symptoms.

## Introduction

Chronic obstructive pulmonary disease (COPD) is a progressive disease associated with substantial morbidity and mortality [1]. It represents a significant economic burden in terms of direct healthcare costs and indirect costs, such as loss of productivity and premature death [2].

Characteristic symptoms of COPD, including progressive dyspnea, cough and sputum production, have a considerable impact on patients’ lives, in particular on those of patients with severe COPD [3]. Breathlessness, which is a consequence of the characteristic lung hyperinflation seen in COPD [4], is one of the most frequently reported symptoms [3] and significantly limits exercise capacity [5]. Several studies have described fluctuations of COPD symptoms over the day, with morning considered the time when symptoms are more severe [3,6-9]. Morning symptoms restrict patients’ ability to carry out morning routines and everyday activities [3,6-8] and might be associated with increased frequency of exacerbations [8].

Two of the main aims of pharmacological management of COPD are relief of symptoms and increase of patients’ ability to exercise and carry out daily activities, with the final goal of improving important outcomes in COPD, such as health-related quality of life [1]. Long-acting inhaled bronchodilators are the recommended first-line maintenance therapy for COPD; treatment options include long-acting muscarinic antagonists (LAMAs) and long-acting β_2_-agonists (LABAs) [1]. According to the new Global Initiative for Chronic Obstructive Lung Disease (GOLD) strategy document, inhaled corticosteroids (ICS) are only recommended in combination with LABAs, in patients with severe and very severe airflow limitation (GOLD severity of airflow obstruction grades 3 and 4) and/or frequent exacerbations (group C and D in the 2011 update). Other guidelines and marketing authorizations restrict the use of these combinations to patients who exhibit both these features and are not controlled by long-acting bronchodilators [1]. Although it has been well established that long-acting bronchodilators are effective in improving COPD symptoms, patients’ health status and ability to exercise [1], data on the specific impact of pharmacological therapies on morning symptoms and activities are still limited.

This review will highlight the impact of COPD symptoms in the morning and provide an overview of tools used to evaluate them. Available evidence on the effect of pharmacological therapies on morning symptoms will also be presented.

### Impact of symptoms in the morning on patients with COPD

**Figure 1:**
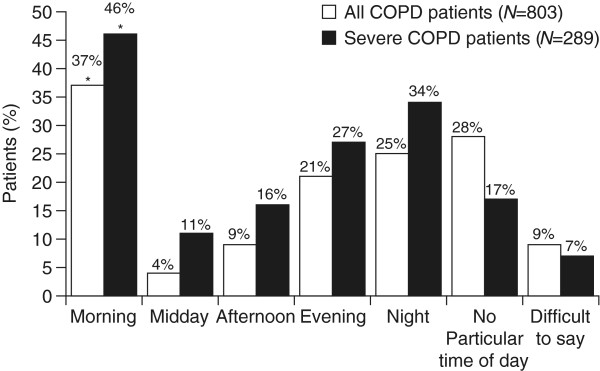
**Time when COPD symptoms are worse than usual as reported by patients.** *p < 0.001 versus midday, afternoon, evening, night and difficult to say group; p = 0.006 versus no particular time of the day; ^†^p < 0.001 versus midday. Copyright © 2009, Informa Healthcare. Reproduced with permission of Informa Healthcare [3].

Circadian variation in lung function has been described in patients with stable COPD, including variation in inspiratory capacity (IC) [10], forced expiratory volume in 1 second (FEV_1_) [10,11], forced vital capacity [10,11], and peak inspiratory flow [12]. Cholinergic tone also has a normal circadian rhythm with higher levels during the sleeping hours, which can lead to airflow limitation in patients with COPD and contribute to the variability of symptoms [11]. An internet survey conducted in 803 patients with COPD revealed that, from a patient’s perspective, morning was the worst time for COPD symptoms, particularly in patients with severe COPD, with shortness of breath being the most frequently reported symptom, followed by sputum production and cough [3]. Overall, the proportion of patients who reported worst symptoms in the morning (37%) was higher than the proportion reporting more pronounced symptoms at any other times of the day (Figure 1). This difference was even more pronounced in patients with severe COPD (46%; p < 0.001; Figure 1). Night was the second most bothersome time with respect to symptoms as reported by 25% of all patients and 34% of patients with severe COPD (Figure 1). However, whether or not a link exists between early morning and night-time symptoms is not yet known.

The survey also revealed that morning symptoms had a significant impact on morning routine and negatively affected patients’ quality of life. Indeed, 74% of all patients and 96% of severe patients reported that they took longer to complete their morning routine than they used to before having COPD. Thirty-seven percent of all patients with COPD and 73% of patients with severe COPD reported their problems with morning routine as bothersome.

**Figure 2:**
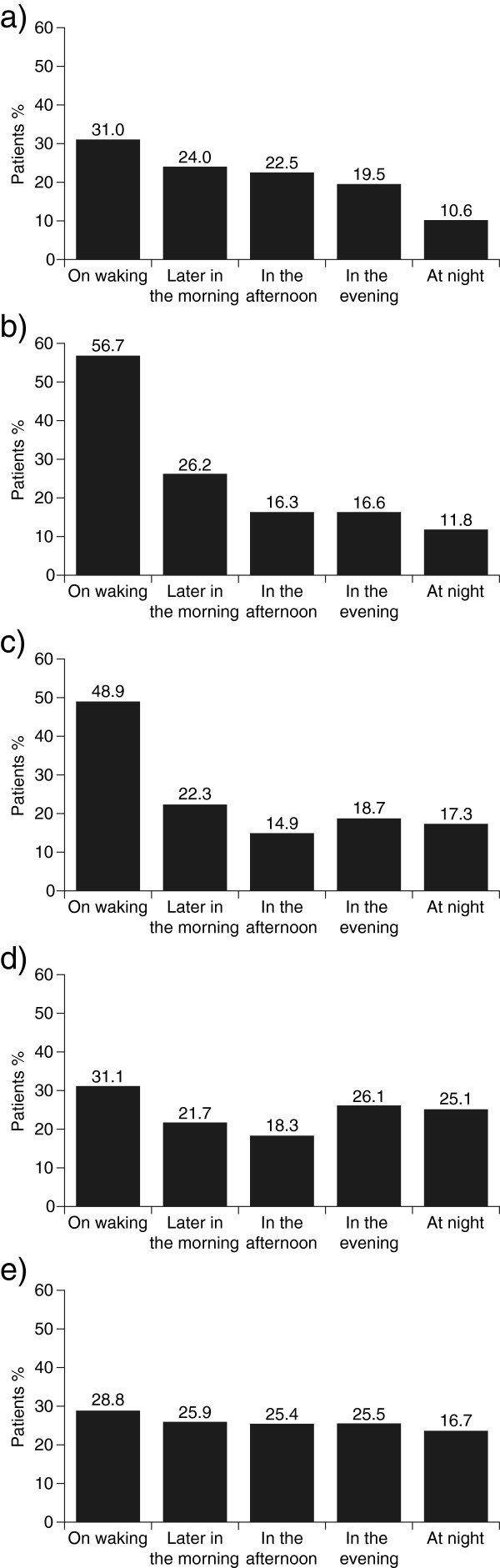
**Patients who had reported experiencing symptoms in the previous 7 days were asked during what times of the day the symptoms were most troublesome: a) Breathlessness, n = 1,769; b) phlegm, n = 1,551; c) cough, n = 1,433; d) wheezing, n = 1,018; and e) chest tightness, n = 690.** © 2002 European Respiratory Society. Reproduced with permission of the European Respiratory Society [7].

Observational studies in large cohorts of patients confirmed that all COPD symptoms were most problematic upon waking [7,13,14]. At this time, the symptoms reported as particularly bothersome by the highest proportion of patients were sputum production (phlegm), followed by cough (Figure 2) [7].

In a study by Kim et al., all COPD symptoms were more frequent and severe in patients with symptoms in the morning than in those without, with symptom scores of breathlessness and chest tightness significantly increased in patients experiencing symptoms in the morning [14]. Interestingly, in this study treatment with a LAMA was associated with fewer morning symptoms.

In addition, the study by Kessler et al. showed that the variability of breathlessness during the course of the day and the week correlated with the severity of this symptom, suggesting that both features are associated with the degree of respiratory impairment and its impact on exercise limitation and daily activity [7]. The variability of breathlessness during the week was also associated with frequent exacerbations (two or more) in the previous year, confirming earlier findings linking symptoms perceived by patients with the rate of exacerbations [9]. Thus, symptoms variability might be of use to identify patients at risk of future exacerbations i.e., belonging to the so-called frequent exacerbator phenotype [15].

More recently, an analysis investigating the impact of symptoms in the morning by self-completion questionnaires in a working COPD population receiving an ICS/LABA fixed-dose combination has further revealed that, in addition to affecting morning activities, these symptoms were associated with an increased risk of exacerbations [8]. Patients with symptoms in the morning had significantly more exacerbations in the previous 12 months than those without (1.04 versus 0.63; p = 0.005). Although no causality was established in this study, these findings suggest that the presence of morning symptoms could be a marker of globally increased airways instability, which could itself be associated with an increased risk of acute worsening. Predictably, patients experiencing symptoms in the morning had a significant wider range of symptoms (3.2 versus 1.6; p < 0.0001) and were 1.73 times more likely to use their rescue inhaler in the previous 4 weeks than those without (p = 0.038) in order to achieve better symptom control [6]. The analysis also revealed that patients with symptoms in the morning took significantly more days off work in the previous 12 months (4.03 versus 2.27; p < 0.01) than those without. This highlights how symptoms in the morning also affect the working lives of patients, contributing to aggravate the economic burden associated with COPD.

In contrast to the growing evidence available on the effect of morning symptoms on different aspects of patients’ lives, the impact of night-time symptoms on long-term patient outcomes in COPD is yet to be established and deserves further investigation.

### PRO questionnaires for the evaluation of morning symptoms

A regular evaluation of symptoms and their variability by validated patient-reported outcome (PRO) questionnaires is vital for assessing the impact of COPD, the effect of pharmacological treatments on morning symptoms and developing effective management strategies. Patient-reported questionnaires traditionally utilized to assess symptoms and health status in patients with COPD, including the St George’s Respiratory Questionnaire (SGRQ) and the Baseline and Transition Dyspnea Index (TDI), do not specifically address the variability of COPD symptoms nor measure the impact of symptoms on important routine morning activities [16]. Significant efforts have been focused on developing validated PRO tools that specifically capture symptoms in the morning and the ability to perform morning activities [17].

The Global Chest Symptoms Questionnaire (GCSQ) and the Capacity of Daily Living during the Morning (CDLM) questionnaire were developed based on interviews with COPD patients and evaluated in two multicenter, randomized trials involving a total of 1,100 COPD patients, with regards to their reliability, validity and responsiveness to therapy [17]. One of the studies evaluated the effect of once-daily tiotropium versus a combination of tiotropium plus twice-daily budesonide/formoterol, while the other one compared the effect of budesonide/formoterol and fluticasone/salmeterol, both inhaled twice daily. Symptoms were assessed by the GSCQ questionnaire whereas morning activities, such as eating breakfast and getting dressed, were assessed using the CDLM questionnaire (Appendix). The responses were recorded in an e-diary. CDLM and GCSQ scores correlated significantly with symptoms, health-related quality of life evaluated by the SGRQ and use of rescue medication (all p < 0.001). In addition, significant improvement in CDLM and GCSQ scores occurred in response to treatment (p < 0.001). Minimal important differences (MIDs) of 0.2 for the CDLM and 0.15 for the GCSQ questionnaires were estimated based on the dataset; these correspond to an SGRQ score MID of 4. Overall, these questionnaires have been shown to be reliable and responsive to treatment effects and could discriminate between patients with different health status [17]. They hold, therefore, significant potential as assessment tools in COPD.

However, these questionnaires may not fully meet new US Food and Drug Administration’s requirements for use in clinical trials to support label claims [18]. In order to overcome this limitation, the Early Morning Symptoms of COPD Instrument (EMSCI), a new PRO tool to evaluate symptoms in the morning, has recently been developed [19]. An electronic version of this tool is currently being tested for use in clinical trials.

Given the prevalence of sleep disturbance associated with night-time symptoms in patients with COPD, new specific instruments have also been recently developed to capture sleep problems [20,21]. A COPD night-time symptoms questionnaire has been specifically designed for Phase III studies with the novel LAMA aclidinium but its robustness and validity for use in clinical trials have not yet been evaluated [22]. In the study, the frequency of night-time symptoms (including breathlessness, cough, sputum production, and wheezing), the severity and impact of breathlessness and cough on night-time activity, and the severity and impact of breathlessness on early morning activity were recorded by patients using a sleep diary [22]. More recently, the COPD and Asthma and Sleep Impact Scale questionnaire has been shown to correlate with the SGRQ questionnaire and discriminate between patients with different severities, exacerbation status and overall health status, suggesting that this tool can help understand the impact of COPD on sleep outcomes [23].

### Controlling morning symptoms in COPD: pharmacological therapies

Although a significant number of studies have shown the effectiveness of pharmacological treatment in decreasing symptoms in patients with COPD, only a few have specifically assessed how these treatments can control early morning symptoms. Several therapeutic options can be considered in COPD when approaching morning symptoms. Better (i.e., more stable) 24-hour coverage provided by once-daily, ultra-long acting bronchodilators is a valuable option for alleviating morning symptoms. Alternatively, better coverage of night-time and early morning can be achieved by twice-daily dosing of long-acting bronchodilators [24-26]. Among long-acting bronchodilators, it might be hypothesized that fast-acting agents could be more effective on these symptoms than those with a relatively slow onset of action by providing a rapid relief of symptoms after morning dosing [27]. Finally, the combination of bronchodilators with different mechanisms of actions, which has been shown to offer advantages over bronchodilator monotherapy in controlling COPD symptoms [28], could potentially offer benefits to patients in the morning.

### Fast-acting LABAs: indacaterol and formoterol

Indacaterol is a once-daily LABA with a fast onset of action, a peak effect at approximately 2 hours and duration of bronchodilation lasting 24 hours [29-31]. Indacaterol has a rapid onset of bronchodilation effect following the first dose. Several different studies have shown that the range of improvement in mean FEV_1_ measured at 5 minutes post-dose was significantly greater with indacaterol than placebo [29,30], approximately double the corresponding value for the slow-acting LABA salmeterol [31], and similar to that for the twice-daily LABA formoterol [29,32] (Table 1). Indacaterol was superior to formoterol in improving breathlessness, measured using the TDI score, and health status assessed using the SGRQ questionnaire, and reducing rescue medication use [29]. The once-daily dosing of indacaterol could also potentially have a compliance-enhancing advantage over the twice-daily formoterol. Further, with its fast onset of action, indacaterol could have significant morning benefits. However, studies are still needed to demonstrate these hypotheses.

**Table 1:** Onset of action of available LABAs and LAMAs

**Agent**	**Dosing regimen,μg**	**Range of increase in FEV**_**1**_**at 5 minutes post**-**dose versus placebo**	**Time to peak bronchodilation**
**LABAs**			
Indacaterol [29,30]	150 q.d. 300 q.d.	110–130 mL (both doses)	Approx. 2 hours
Salmeterol [31]	50 b.i.d.	60 mL	Approx. 2 hours
Formoterol [29,32]	12 b.i.d. 24 b.i.d	120–140 mL (both doses)	Approx. 2 hours
**LAMAs**			
Aclidinium [34]	200 b.i.d 400 b.i.d	Approx. 70 mL Approx. 105 mL	Approx. 2 hours
Tiotropium [30,42]	18 q.d.	45–70 mL	Approx. 2 hours
Glycopyrronium [42]	50 q.d	87 mL	Approx. 2 hours

b.i.d = twice daily; *FEV*_1_ = forced expiratory volume in 1 second; *LABAs* = long-acting β2-agonists; *LAMAs* = long-acting muscarinic antagonists; q.d. = once daily.

The effect of formoterol in combination with ICS on COPD symptoms in the morning has been evaluated in two independent studies. In a randomized, double-blind, double-dummy, cross-over study, budesonide/formoterol dosed in the morning demonstrated a more rapid onset of action than salmeterol/fluticasone, as shown by significantly greater improvements in FEV_1_ at 5 minutes post-dose (120 mL versus 90 mL; p = 0.09) and 15 minutes post-dose (140 mL versus 100 mL; p < 0.05) [33]. Accordingly, treatment with budesonide/formoterol resulted in a statistically significant improvement in total score on CDLM questionnaire compared with salmeterol/fluticasone. These advantages are likely to be related to the fast onset of action of formoterol versus salmeterol post-dose. Their clinical relevance needs to be explored further.

### New fast-acting LAMAs: aclidinium and glycopyrronium

Aclidinium, a novel twice-daily LAMA, has recently been approved as a maintenance treatment for COPD. Phase II and Phase III studies, have shown aclidinium improved lung function, dyspnea, health status and exercise endurance and was well tolerated in patients with moderate-to-severe COPD [34-37]. In a direct comparison, aclidinium did not display a faster onset of action on bronchodilation than tiotropium [38]. However, as aclidinium is administered twice daily, it has been hypothesized that there could be less tail-off effect in the morning, which could be associated with an increased overall effect on late night and early morning symptoms. Indeed, in the study by Fuhr et al. aclidinium resulted in improved bronchodilation at night versus tiotropium, as demonstrated by significantly greater improvement from baseline in FEV_1_ area under the curve (AUC)_12–24 hours_, which translated in significantly improvement in night-time symptoms [25]. Further studies have shown that aclidinium twice daily improves symptoms (cough and breathlessness) in the morning/early morning and during night-time [22,37,39]. However, effects on morning symptoms were not compared to overall effect on symptoms, which precludes any conclusion regarding a “specific” effect on morning symptoms versus other long-acting bronchodilators. Thus, the positive impact of twice-daily aclidinium on COPD symptoms at night-time and in the early morning still needs to be confirmed using validated PRO questionnaires.

The once-daily LAMA glycopyrronium has also recently been approved for patients with COPD. Phase II and III clinical trials have shown that once-daily glycopyrronium produces rapid and sustained 24-hour bronchodilation in patients with COPD, and has an acceptable safety and tolerability profile [40-44]. Glycopyrronium also decreases symptoms (i.e., sputum production and dyspnea) and the use of rescue medication, a marker of symptom control, and frequency of exacerbation, and improves health status [40,42]. In the GLOW3 study, glycopyrronium rapidly improved exercise tolerance by reducing dynamic hyperinflation, as shown by the significant increase in IC at isotime versus placebo on Day 1 (treatment difference: 230 mL; p < 0.001) [45].

In Phase III studies, once-daily glycopyrronium taken in the morning displayed a rapid onset of action on Day 1 which was faster than that of tiotropium [42], and produced a greater increase in FEV_1_ at 5 minutes post-dose (Table 1) and greater peak FEV_1_ and FEV_1_ AUC_0–4 hours_ than placebo and tiotropium at Day 1 and Week 26 [42]. This early bronchodilation following morning dose, could in turn provide quicker relief from symptoms in the morning, such as shortness of breath, which hinder patients’ ability to perform simple morning activities. However, its effect on symptoms experienced in the morning has not yet been formally evaluated with validated questionnaires.

### The potential of dual bronchodilation in controlling morning symptoms

Despite the symptomatic benefits that could be provided by dual bronchodilation to patients with COPD [28], evidence for its impact on morning symptoms is still scarce. It can be speculated that combining treatments from different classes should result in additional efficacy. Studies with the dual bronchodilator QVA149, a fixed-dose combination of indacaterol and glycopyrronium, show improvements in lung function and symptoms with the combination compared with the individual monocomponents used alone [46]. The effect of QVA149 on morning symptoms is currently under investigation in clinical trials [47]. Further, in a 12-week, randomized, double-blind study budesonide/formoterol added to tiotropium was shown to rapidly improve lung function (mean treatment difference for FEV_1_ at 5 minutes post-dose 123 mL; p < 0.001) compared with tiotropium alone, mainly owing to the fast onset of action of formoterol [48]. This was associated with significant improvements in morning symptoms, including breathlessness, as evaluated by the CDLM questionnaire, as well as increased patients’ ability to perform morning activities. However, as the study did not include a tiotropium plus formoterol arm, it is difficult to discern the contribution of pure dual bronchodilation to the overall results and further studies are warranted. Nevertheless, the results of the study suggest that a fast-acting component might be useful to help control morning symptoms in patients on LABA/ICS treatment.

## Conclusions

COPD symptoms in the morning are strongly associated with problems experienced by patients in performing simple morning activities, resulting in a noticeable impact on patients’ quality of life. These symptoms, which are particularly pronounced in patients with severe COPD, often result in work absenteeism therefore contributing to aggravate the already substantial economic burden imposed by COPD.

In light of this consideration, the development of validated PRO tools able to capture the experience of patients with regards to symptoms in the morning becomes vital for evaluating the impact of this disease on patients’ lives and establishing effective and comprehensive management strategies. A number of available PRO questionnaires have already been used to assess the effect of therapeutic interventions on symptoms in the morning. The CDLM and GCSQ questionnaire have been utilized in two clinical trials evaluating the effect of the budesonide/formoterol fixed-dose combination versus combination therapy with salmeterol/fluticasone or budesonide/formoterol added to the LAMA tiotropium versus tiotropium alone. Budesonide/formoterol provided a better control of symptoms in the morning than salmeterol/fluticasone, which was associated with a positive effect on morning activities. Similar results were obtained with budesonide/formoterol plus tiotropium compared with tiotropium alone. In both studies, a rapid improvement of morning lung function, which was the result of the fast onset of action of formoterol, was associated with positive outcomes with regards to symptoms in the morning. This highlights the potential that other fast-acting bronchodilators could have in this context.

The newly approved twice-daily LAMA aclidinium has been shown to significantly and rapidly improve morning lung function as well as reduce symptoms at night-time and in the early morning. As this effect of aclidinium was assessed by a non-validated questionnaire specifically developed for these studies, whether the same results will be obtained with validated tools still remain to be assessed.

The new once-daily LAMA glycopyrronium displays a faster onset of action and greater early bronchodilation than tiotropium following morning dosing, and could have the potential to reduce COPD symptoms in the morning, enabling patients to cope with morning activities. Evaluation of its effect on COPD symptoms in the morning is needed.

Although reliable and responsive, many of the PRO questionnaires validated in clinical trials so far have been designed before new regulatory guidelines and could not be used to support labeling claims. The ongoing collaboration between regulatory bodies, academia and the pharmaceutical industry is driving the development of standardized instruments able to satisfy regulatory requirement, such as the EMSCI tool. Such tools could be consistently used to evaluate the success or failure of therapeutic interventions throughout the drug development process, therefore allowing the comparison between different agents.

In conclusion, we believe that given the importance of morning symptoms and the existence of validated instruments, the impact of morning symptoms should be evaluated in future clinical trials, particularly focusing on their relationship with long term outcomes and response to treatment effects.

## Appendix. Patient-reported outcomes questionnaire

A. **Global Chest Symptoms questionnaire**

1. How short of breathe are you feeling right now?

1. How tight does your chest feel right now?

B. **Capacity of Daily Living during the Morning questionnaire**

1. Did you wash yourself this morning other then your face?

1. Did you dry yourself with a towel after washing this morning?

1. Did you get dressed this morning?

1. Did you eat breakfast this morning

1. Did you walk around the home this morning after taking your medicine?

1. Did you walk around d your home later this morning?

[A] Patient responses were: not at all, a little, moderately, very or extremely.

[B] If the patient answered 'Yes, I did it myself’ (question 1 − 3) or 'Yes, I did' (question 4 − 6)

The question was followed by 'How difficult was it to complete the activity?' (patient responses: not at all, a little, moderately, very or extremely).

Each time was scored on a scale ranging from 0 (so difficult that the activity could not be carried out by the patients themselves) to 5 (activity not all difficult to catty out).

## Abbreviations

CDLM: Capacity of Daily Living during the Morning questionnaire; COPD: Chronic obstructive pulmonary disease; EMSCI: Early Morning Symptoms of COPD Instrument; FEV1: Forced expiratory volume in 1 second; GCSQ: Global Chest Symptoms Questionnaire; GOLD: Global Initiative for Chronic Obstructive Lung Disease; IC: Inspiratory capacity; ICS: Inhaled corticosteroids; LAMA: Long-acting muscarinic antagonists; LABA: Long-acting β_2_-agonists; MIDs: Minimal important differences; PRO: Patient-reported outcome; SGRQ: St George’s Respiratory Questionnaire; TDI: Transition Dyspnoea Index.

## Competing interests

In the past 5 years, Nicolas Roche received fees for speaking, organizing education, or consulting from Aerocrine, Almirall, Altana Pharma-Nycomed, AstraZeneca, Boehringer Ingelheim, Chiesi, GlaxoSmithKline, MEDA, MSD-Chibret, Mundipharma, Novartis, Pfizer, Teva; and research grants from Novartis, Nycomed and Boehringer Ingelheim. NHC has received consultancy fees from Pfizer, Boehringer Ingelheim, AstraZeneca, Novartis and Chiesi and research grants from Pfizer, Boehringer Ingelheim, Novartis, Mundipharma and GlaxoSmithKline. MM has received speaker fees from Boehringer Ingelheim, Pfizer, AstraZeneca, Bayer Schering, Novartis, Talecris-Grifols, Takeda-Nycomed, Merck, Sharp & Dohme and Novartis, and consulting fees from Boehringer Ingelheim, Pfizer, GSK, AstraZeneca, Bayer Schering, Novartis, Almirall, Merck, Sharp & Dohme, Talecris-Grifols and Takeda-Nycomed.

## Authors’ contributions

All authors were involved in the concept and design of this article. All authors revised the article critically for important intellectual content and gave their final approval of the version to be published.
